# Accuracy of Xpert Ultra in Diagnosis of Pulmonary Tuberculosis among Children in Uganda: a Substudy from the SHINE Trial

**DOI:** 10.1128/JCM.00410-20

**Published:** 2020-08-24

**Authors:** Willy Ssengooba, Jean de Dieu Iragena, Lydia Nakiyingi, Serestine Mujumbi, Eric Wobudeya, Robert Mboizi, David Boulware, David B. Meya, Louise Choo, Angela M. Crook, Kristen Lebeau, Moses Joloba, Anne-Marie Demers, Fiona V. Cresswell, Diana M. Gibb

**Affiliations:** a Makerere University, Department of Medical Microbiology, Mycobacteriology (BSL-3) Laboratory, Kampala, Uganda; b Makerere University Lung Institute, Makerere University College of Health Sciences, Kampala, Uganda; c Communicable Diseases Cluster, HIV/TB and Hepatitis Programme, World Health Organization Regional Office for Africa, Brazzaville, Congo; d Infectious Diseases Institute, College of Health Sciences Makerere University, Kampala, Uganda; e Makerere University-Johns Hopkins University Research Collaboration, Kampala, Uganda; f Clinical Research Department, London School of Hygiene and Tropical Medicine, London, United Kingdom; g MRC-UVRI LSHTM Uganda Research Unit, Entebbe, Uganda; h MRC Clinical Trials Unit, University College London, London, United Kingdom; i Desmond Tutu Tuberculosis Centre, Department of Paediatrics and Child Health, Faculty of Medicine and Health Sciences, Stellenbosch University, Cape Town, South Africa; UNC School of Medicine

**Keywords:** accuracy, childhood, diagnosis, tuberculosis, Xpert Ultra

## Abstract

Childhood tuberculosis (TB) presents significant diagnostic challenges associated with paucibacillary disease and requires a more sensitive test. We evaluated the diagnostic accuracy of Xpert MTB/RIF Ultra (Ultra) compared to other microbiological tests using respiratory samples from Ugandan children in the SHINE trial. SHINE is a randomized trial evaluating shorter treatment in 1,204 children with minimal TB disease in Africa and India. Among 352 samples and one cervical lymph node fine needle aspirate, one sample was randomly selected per patient and tested with the Xpert MTB/RIF assay (Xpert) and with Lowenstein-Jensen medium (LJ) and liquid mycobacterial growth indicator tube (MGIT) cultures. We selected only uncontaminated stored sample pellets for Ultra testing. We estimated the sensitivity of Xpert and Ultra against culture and a composite microbiological reference standard (any positive result). Of 398 children, 353 (89%) had culture, Xpert, and Ultra results. The median age was 2.8 years (interquartile range [IQR], 1.3 to 5.3); 8.5% (30/353) were HIV infected, and 54.4% (192/353) were male. Of the 353, 31 (9%) were positive by LJ and/or MGIT culture, 36 (10%) by Ultra, and 16 (5%) by Xpert. Sensitivities (95% confidence intervals [CI]) were 58% (39 to 65% [18/31]) for Ultra and 45% (27 to 64% [14/31]) for Xpert against any culture-positive result, with false positives of <1% and 5.5% for Xpert and Ultra. Against a composite microbiological reference, sensitivities were 72% (58 to 84% [36/50]) for Ultra and 32% (20 to 47% [16/50]) for Xpert. However, there were 17 samples that were positive only with Ultra (majority trace). Among children screened for minimal TB in Uganda, Ultra has higher sensitivity than Xpert. This represents an important advance for a condition which has posed a diagnostic challenge for decades.

## Abstract (precis)

Childhood tuberculosis (TB) presents significant diagnostic challenges associated with paucibacillary disease and requires a more sensitive test. We evaluated the diagnostic accuracy of Xpert MTB/RIF Ultra (Ultra) compared to other microbiological tests using respiratory samples from Ugandan children in the SHINE trial. SHINE is a randomized trial evaluating shorter treatment in 1,204 children with minimal TB disease in Africa and India. Among 352 samples and one cervical lymph node fine needle aspirate, one sample was randomly selected per patient and tested with the Xpert MTB/RIF assay (Xpert) and with Lowenstein-Jensen medium (LJ) and liquid mycobacterial growth indicator tube (MGIT) cultures.

## INTRODUCTION

More than 96% of tuberculosis (TB)-related pediatric deaths are estimated to occur in children not receiving antituberculosis treatment (1), highlighting the importance of early and accurate diagnosis. Mycobacteriological diagnostics used for adults are less sensitive for samples from children (2), due to the paucibacillary nature of childhood TB disease and the difficulties of obtaining adequate specimens for bacteriological confirmation, as young children are frequently unable to voluntarily expectorate sputum (3). The development of rapid and accurate diagnostic TB tests is recognized as a vital part of the WHO End TB Strategy (4), as well as being important to allow appropriate early diagnosis and initiation of TB treatment in children.

In March 2017, Cepheid (Sunnyvale, CA, USA), with endorsement from the World Health Organization (WHO), launched the next-generation Xpert MTB/RIF Ultra assay (referred to here as Ultra) (5, 6), with the aim of further increasing diagnostic sensitivity. Two significant changes were made along with other technical optimizations. First, each cartridge includes a larger chamber for DNA amplification (50 μl compared to 25 μl), thus accommodating twice the volume of sample for the PCR. Second, two additional multicopy molecular targets for Mycobacterium tuberculosis, IS1081 and IS6110, were introduced, alongside 4 *rpoB* gene probes, resulting in a decrease in the limit of detection (LOD) *in vitro* from ∼113 bacilli per ml of sputum for Xpert to ∼16 for Ultra, with a trace category added for lowest bacillary load (7). Ultra runs on the same GeneXpert platform as Xpert MTB/RIF (using software version 4.7b or later) as well as on the GeneXpert Omni platform.

A multicenter noninferiority study at ten sites in eight low- and middle-income countries in adults with suspected pulmonary TB found that Ultra was significantly more sensitive than Xpert against a culture-based reference standard in smear-negative TB (63% versus 46%) and HIV-associated pulmonary TB (PTB) (90% versus 77%), but with more modest gains in the general population (88% versus 83%) (7). Five additional studies have evaluated Ultra in adult populations (6–9). To date, only two studies have evaluated the accuracy of Ultra for the diagnosis of PTB in children suspected of having TB. In a study on 367 biobanked sputum samples in Cape Town, of those with microbiologically confirmed TB (by composite of MGIT [mycobacterial growth indicator tube] culture, Xpert, or Ultra), the sensitivities of culture, Ultra, and Xpert compared to composite reference were 83%, 74%, and 62%, respectively (10). In a further study of 215 children, using clinical case definition as the reference standard, the sensitivity was 64% for Ultra compared to 54% for Xpert (11).

There remains a paucity of data evaluating the performance of Ultra in childhood PTB. Considering that culture is expensive and slow, may be prone to contamination, and is not always readily available in resource-constrained settings, the availability of a highly sensitive molecular point-of-care test might allow more rapid TB diagnosis and reduced use of culture techniques in special scenarios such as those with negative molecular tests or suspected drug resistance.

The challenge is how to accurately assess this novel diagnostic assay in the absence of a perfect gold standard and in a population where obtaining high-quality samples is inherently difficult. The culture-based reference standard is widely acknowledged to be imperfect, with results in true childhood TB cases being frequently misclassified; culture techniques can miss up to 40% of childhood pulmonary TB cases (12). Such misclassification by the reference standard makes it difficult to evaluate any novel assay, as true TB cases detected only by the novel assay maybe incorrectly labeled as false positive, thus wrongly reducing the specificity of the novel test.

The aim of this study was to investigate the diagnostic performance of Ultra in children with suspected minimal pulmonary TB disease using cryopreserved respiratory specimens (sputum sediment or gastric aspirate) or a cervical lymph node fine needle aspirate (FNA). In light of the complexities of evaluating diagnostic accuracy in the absence of a perfect gold standard, we used both a culture-based reference standard and a composite microbiological reference standard.

## MATERIALS AND METHODS

### Study design and population.

This was a nested diagnostic substudy using stored specimens from the Ugandan site of the SHINE clinical trial between July 2016 and July 2018. SHINE (shorter treatment for minimal TB in children) is a randomized trial of therapy shortening for minimal tuberculosis with new WHO-recommended fixed-dose-combination drugs in African and Indian HIV-positive and HIV-negative children (International Standard Randomised Controlled Trials no. ISRCTN63579542) (13). Minimal TB in children is defined as a form of nonsevere, symptomatic, smear-negative TB, including extrathoracic lymph node TB, intrathoracic uncomplicated lymph node TB, and other nonsevere forms of pulmonary TB as per an established classification system (14). The trial is evaluating 4 versus 6 months of standard treatment for children with nonsevere drug-susceptible minimal TB disease. Details of the study design and population were described previously (13). Symptomatic children who provided samples in the trial screening process but were not subsequently enrolled in the clinical trial were also eligible for inclusion in this substudy. At least 2 respiratory samples were collected (gastric aspirate or washing, expectorated sputum, or induced sputum) or an FNA. All children enrolled in the trial were subsequently randomized to receive TB treatment of either 4 or 6 months’ duration.

### Laboratory procedures.

Sputum samples collected from participating clinics were delivered the same day under refrigerated conditions to the College of American Pathologists (CAP) ISO 15189-accredited Mycobacteriology Laboratory (biosafety level 3 [BSL-3]) at Makerere University, Kampala, Uganda. Gastric samples were neutralized with bicarbonate at the bedside. Samples were processed using equal volumes of sodium hydroxide (NaOH), sodium citrate, *N*-acetyl-l-cysteine decontaminating solution (NaOH final concentration, 1.5%), vortexed, and incubated for 15 min at room temperature. The samples were neutralized using phosphate-buffered saline (PBS; pH 6.8) and then centrifuged at 3,000 × *g* in a refrigerated centrifuge for 15 min. The supernatant was then decanted, the pellet was suspended in 2.5 ml of PBS, and then 0.5 ml was inoculated into the mycobacterial growth indicator tube (MGIT) and 200 μl each onto two Lowenstein-Jensen (LJ) tubes. One drop, ∼50 μl, of the processed sample was used for fluorescent smear microscopy. One milliliter was used for Xpert testing following the manufacturer’s recommendations, and the remaining pellet was stored at −80°C until use. The MGIT tubes were incubated for up to 6 weeks until growth and LJ tubes for 8 weeks with weekly examination for up to 8 weeks until growth. The growth was identified as M. tuberculosis using a rapid MPT64 antigen test (SD-Bioline; Standard Diagnostics, Yongin-si, Gyeonggi-do, Republic of Korea). All MGIT-negative cultures were visually inspected for growth, and for any suspicious growth, a smear for Ziehl-Neelsen (ZN) staining was done; otherwise, the tubes were discarded. For the current study, we randomly selected one sample with a valid Xpert test and at least one culture which was not contaminated or not growing nontuberculous mycobacteria (NTM) for Xpert Ultra testing.

The selected sputum pellets were retrieved from the freezer and brought to room temperature for testing. Sample reagents (SR) were added to the sample at a ratio of 2:1 (2 parts SR to 1 part sample) according to the manufacturer’s recommendations. Each sample was vortexed and allowed to sit for 20 min before being tested on the Cepheid GeneXpert platform using Ultra cartridges.

### Statistical analysis.

Data were analyzed for frequencies and proportions of participants diagnosed for TB by each test used. We calculated the sensitivity of Xpert Ultra using two reference standards: (i) a culture-based reference standard (MGIT and/or LJ positive for Mycobacterium tuberculosis) and (ii) a composite reference standard of any positive test (Xpert, culture, or Ultra). Our rationale for including Ultra in the composite reference standard is that it is a specific DNA-based test and the likelihood of a false-positive result is likely to be low in a symptomatic child clinically treated for TB in a country with high TB incidence, especially in a young child never previously diagnosed with or treated for TB. In this study, all children were treated for TB and so had met a threshold of clinical suspicion (15).

### Ethical considerations.

The study received ethics committee approval from the Mulago Hospital Research and Ethics Committee and the Uganda National Council of Science and Technology. All participants consented to having their leftover samples stored for future use.

## RESULTS

**FIG 1:**
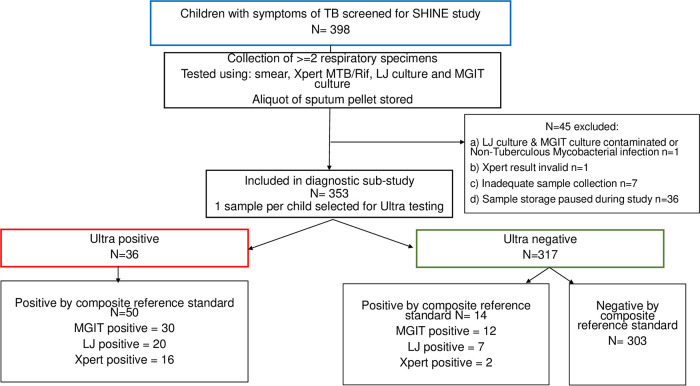
STARD (standards for reporting diagnostic accuracy) flowchart of the Ultra substudy. Xpert, Xpert MTB/RIF; Ultra, Xpert MTB/RIF Ultra; LJ, Lowenstein-Jensen medium culture; MGIT, mycobacterial growth indicator tube culture.

A total of 398 children with suspected TB were eligible for the SHINE trial in Uganda. Of these, 353 children with LJ or MGIT cultures that were not contaminated and not identified as NTM and with valid Xpert results were included in this substudy (Fig. 1). The median age (interquartile range [IQR]) was 2.8 years (IQR, 1.2 to 5.3); 192 (54%) were male, and 30 (8.5%) were HIV positive. Demographics and clinical details are not described in further detail here, as the trial is still ongoing. Respiratory samples included 262 (74.2%) from gastric lavage, 83 (24.5%) sputum samples, 7 (2.0%) gastric washing samples, and one cervical lymph node fine needle aspirate.

### Distribution of TB test results among participants.

**FIG 2:**
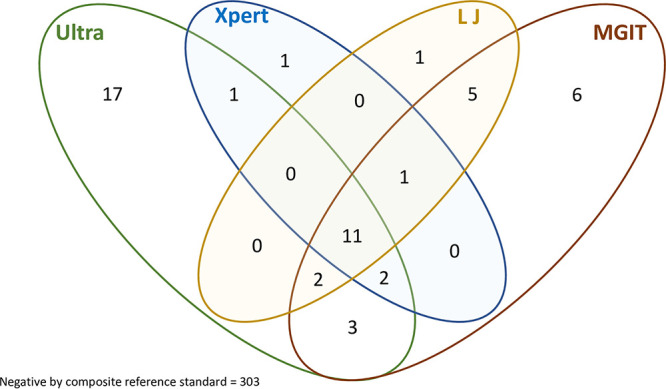
Venn diagram showing proportion of patients diagnosed by each test and a combination of tests (*n* = 353). Xpert, Xpert MTB/RIF; Ultra, Xpert MTB/RIF Ultra; LJ, Lowenstein-Jensen medium culture; MGIT, mycobacterial growth indicator tube culture.

Fifty (14.2%) of the participants had one or more positive test, including 16 (4.5%) by Xpert, 20 (6.0%) by LJ culture, 30 (9.2%) by MGIT culture, and 36 (10.2%) by Ultra. Six participants were exclusively positive by MGIT, one by Xpert, and one by LJ culture (Fig. 2). Ultra was positive for 29/36 (11.1%) gastric lavage and 7/36 (8.4%) induced sputum samples (*P* = 0.704). Of the 13 Ultra-negative but culture-positive samples, 12 were gastric lavage fluid and only one was sputum. Seventeen patients’ samples were positive exclusively by Ultra, of which 16 (94.1%) were positive in the trace semiquantitative category, suggestive of a very low bacillary load, and 1 (5.9%) was very low.

Ultra semiquantitative trace category results give a rifampin resistance result of “indeterminate” due to the low quantity of DNA present, precluding assessment for rifampin resistance-conferring mutations in the *rpoB* gene. Of those with informative rifampin results, none had resistance to rifampin by Xpert or Ultra.

### Ultra semiquantitative category and days to culture positivity.

In terms of Ultra semiquantitative categories, Ultra positivity was reported as medium for 2 samples (5.6%), low for 7 (19.4%), very low for 7 (19.4%), and trace for 20 (55.6%). Culture was positive among all samples reported as medium, 6/7 reported as low, 6/7 reported as very low, and 4/20 reported as trace (Table 1). Median time (days) to culture positivity by Ultra semiquantitative category was 11 days (IQR, 10 to 11.0) for the positives in the medium category, 9 days (8 to 11.0) for the low category, 11 (9 to 16) for the very low category, and 15 (12 to 18) for the trace category.

**TABLE 1:** Ultra semiquantitative category and days to MGIT culture positivity^*a*^

SQ category	Xpert (*n* = 16)	Ultra (*n* = 36)	No. of days to MGIT culture positivity by Ultra SQ category	
			No. (*n* = 18)	Median (IQR)
High	0	0	0	NA
Medium	2	2	2	11 (10–11)
Low	5	7	6^*b*^	9 (8–11)
Very low	9	7	6^*b*^	11 (9–16)
Trace	NA^*c*^	20	4^*b*^	15 (12–18)

a SQ, semiquantitative; IQR, interquartile range; Xpert, Xpert MTB/RIF; Ultra, Xpert MTB/RIF Ultra; MGIT, mycobacterial growth indicator tube.

b Other samples were MGIT culture negative.

c NA, the trace category is not reported by Xpert.

Among the 20 Ultra positive results in the trace category, 17 had no growth and three had colony counts of 10 to 100 on LJ culture. On MGIT culture, 13 samples had no growth, three were contaminated, and four were positive at 12, 13, 17, and 18 days of incubation.

### Diagnostic accuracy by both a culture-based reference standard and a composite microbiological reference standard.

A total of 31/353 (9%) samples were positive by culture, including 20/335 (6.0%) and 30/325 (9.2%) that were positive by LJ and MGIT cultures, respectively. When culture was used as the reference standard, the sensitivities were 45.2% (14/31; 95% confidence interval [CI], 27.3 to 63.9) for Xpert and 58.1% (18/31; 95% CI, 39.0 to 75.4) for Ultra. Using a composite microbiological reference standard, the sensitivities were 32.0% (16/50; 95% CI, 19.5 to 46.6) for Xpert, 72.0% (36/50; 95% CI, 57.5 to 83.7) for Ultra, 42.6% (20/47; 95% CI, 28.2 to 57.8) for LJ culture, and 63.8% (30/47; 95% CI, 48.5 to 77.3) for MGIT culture (Table 2). In terms of specificity, against the culture-based reference Xpert had a specificity of 99.4% (320/322; 95% CI, 97.7 to 99.9) and Ultra had a specificity of 94.4% (304/322; 95% CI, 91.3 to 96.6).

**TABLE 2:** Diagnostic accuracy of Ultra compared to culture for childhood TB diagnosis^*a*^

Test	% (95% CI), *n*/*N*			
	Sensitivity	Specificity	PPV	NPV
Culture-based reference standard				
Xpert	45.2 (27.3–63.9), 14/31	99.4 (97.7–99.9), 320/322	87.5 (61.6–98.4), 14/16	95.0 (92.0–97.0), 320/337
Ultra	58.1 (39.0–75.4), 18/31	94.4 (91.3–96.6), 304/322	50.0 (32.9–67.0), 18/36	95.9 (93.0–97.7), 304/317
Composite microbiological reference standard				
Xpert	32.0 (19.5–46.6), 16/50	100, 303/303	100, 16/16	89.9 (86.1–92.9), 303/337
Ultra	72.0 (57.5–83.7), 36/50	100, 303/303	100, 36/36	95.6 (92.7–97.5), 303/317
LJ culture	42.6 (28.2–57.8), 20/47^*b*^	100, 288/288	100, 20/20	91.4 (87.7–94.2), 288/315
MGIT culture	63.8 (48.5–77.3), 30/47^*b*^	100, 278/278	100, 30/30	94.2 (90.9–96.6), 278/295

a Xpert, Xpert MTB/RIF; Ultra, Xpert MTB/RIF Ultra; LJ, Lowenstein-Jensen medium; MGIT, mycobacterial growth indicator tube; CI, 95% confidence interval; PPV, positive predictive value; NPV, negative predictive value; *n*/*N*, number positive/total number.

b Three samples were contaminated by the indicated method.

## DISCUSSION

This diagnostic substudy among children clinically treated for minimal TB disease at the Ugandan site of the SHINE trial found that 50/353 (14.2%) of children had microbiologically confirmed TB when samples positive only by Ultra were treated as true positives. When samples positive only by Ultra were excluded, the prevalence of microbiologically confirmed TB was 33/353 (9.4%).

There was a surprisingly high number of samples (*n* = 17) positive only by Ultra, almost all of which were positive in the trace category. There are a number of possible explanations for this finding, and it is important to interpret this in the context of this specific study population of children (98% with minimal TB disease [i.e., all sputum smear negative]); the yield of all tests used would be lower than expected for children with sputum smear-positive TB. The smear-negative nature of this study population may affect some tests to a greater degree than others. Studies using spiked sputum samples have shown that around half of samples are positive by Ultra with as few as 2.5 CFU/ml (5, 6). Thus, it is possible that samples positive only by Ultra may have had a bacillary load too low to be detectable by Xpert (LOD, ∼116 CFU/ml) and potentially also too low for culture (LOD, >10 CFU/ml). Another explanation is that bacilli were rendered nonviable (and thus culture negative) by gastric acid, by delays in inoculation of the culture medium, by antituberculosis therapy initiated before the time of testing, or by host inflammatory response. In a recent study evaluating the diagnostic value of Ultra in childhood tuberculosis using high-quality respiratory samples (bronchoalveolar lavage fluid), 58% of Ultra-positive samples were negative on both smear microscopy and culture (16).

Conversely, one could view the samples that were positive only by Ultra as false positives. A number of adult studies have shown that MTB DNA can be detected in sputum by Ultra (usually trace category positive) in subclinical (incipient) TB and also years after completion of “effective” antituberculosis therapy for prior pulmonary tuberculosis (17). To our knowledge there are no data on duration of Ultra positivity in children, but we do not feel this to be a plausible explanation in our study, as the median age of the children was 2.8 years (IQR, 1.3 to 5.3) and none of the children who were Ultra trace positive only had had prior PTB diagnosis or treatment. The possibility of false-positive results would also be more plausible for a child who was asymptomatic or from a low-prevalence area. In this study setting, all children had sufficient signs and symptoms predominantly for pulmonary TB to warrant initiation of anti-TB therapy by the study doctor. Moreover, the WHO Technical Expert Group recommended that among persons with HIV, children, and extrapulmonary specimens, “trace calls” should be considered to be true-positive results for use in clinical decisions and patient follow-up (17).

It is, however, curious that Ultra could be sensitive enough to pick up culture-negative TB but give negative results for a total of 13 samples that were positive by culture (LJ and/or MGIT) (Fig. 2). This finding could relate to sample processing. Samples were centrifuged, and then the cell pellet was resuspended in 2.5 ml PBS; unless the sample was well vortexed, creating a homogenous suspension, the bacilli could clump, and a greater number could be aliquoted into one test sample (the smear, Xpert, LJ culture, MGIT culture, or storage sample). Additionally, storing of an aliquot for a prolonged period (up to 2.5 years for early samples) could have negatively impacted the yield of the sample. However, on analysis of cases diagnosed by culture, 4/9 (44.4%) in 2016, 8/13 (61.5%) in 2017, and 1/9 (11.1%) in 2018 were discordant, suggesting no clear trend of discordance related to sample storage duration, although discordance was lower in more recent samples. Gastric lavage samples may perform less well than sputum on Ultra, as suggested by a recent study (18); the majority of samples in our study were gastric lavage fluid, and among the 13 Ultra-negative but culture-positive samples, 12 were gastric lavage fluid. Studies have documented inconsistent yields with different sample types collected for childhood TB diagnosis (19, 20), and this needs further investigation. However, the degree of discordance between DNA tests and culture highlights the fact that there remains a role for respiratory specimen cultures in children with suspected TB.

In light of the lack of a gold standard against which to evaluate the performance of Ultra, we used two reference standards, each susceptible to different forms of bias. Firstly, against the culture-based reference standard, Ultra has a higher sensitivity than Xpert MTB/RIF (58% versus 45%; *P* = 0.309 [nonsignificant]), and the specificity of Ultra was 94%, potentially relating to misclassification bias. A similar-sized study on stored samples from children in Cape Town yielded comparable sensitivity results against a culture-based reference (sensitivities of 74% for Ultra and 64% for Xpert, and a specificity of 97.2% for Ultra), adding weight to the validity of our findings, though they had fewer cases that were positive only by Ultra (*n* = 8).

The second reference standard used, the composite microbiological reference standard, risks incorporation bias by including the index test (Ultra) in the reference standard. Against a composite microbiological reference standard, the sensitivity of Ultra was superior to that of Xpert (72% versus 32%; *P* < 0.001) and LJ culture (72% versus 43%; *P* = 0.003) and nonsignificantly better than that of MGIT (72% versus 64%; *P* = 0.389).

The lack of clarity about the status of the trace-positive Ultra samples needs further investigation; we plan to do this by using data from all 1,204 children in the trial, including baseline clinical, radiological, and microbiologic data, when these are available for analysis at the end of the trial.

Ultra’s diagnostic sensitivity and ability to detect rifampin resistance, its turnaround time of 84 min, and the widespread availability of the Xpert platform in the public sector in low- and middle-income countries provide support for Ultra potentially being a best initial test for childhood PTB in such settings. Many countries are now transitioning from Xpert to Ultra, and the Xpert platform had been rolled out in 130/145 countries eligible for concessional pricing by 2016. WHO and the Foundation for Innovative New Diagnostics (FIND) have analyzed existing evidence and believe that for children and people living with HIV, the benefits of Ultra’s increased sensitivity outweigh the potential harm and cost of decreased specificity. It is interesting that our study showed that performing Ultra followed by liquid culture by MGIT identified 95% (49/50) of pediatric TB cases, which further indicates the value of liquid over solid culture in childhood TB diagnosis.

Strengths of this study include the large sample size, making it one of the larger studies of Ultra in childhood PTB to date, although the findings cannot necessarily be generalized outside the population in which it was studied, (i.e., those with minimal TB disease). Ultra may perform differently in different populations. All diagnostic testing for this trial was conducted in a College of American Pathologists (CAP) ISO 15189-accredited BSL-3 mycobacteriology laboratory, so the possibility of sample contamination or processing errors is potentially reduced. Limitations include the lack of baseline clinical data and outcome data for those who tested positive only with Ultra, as these data have yet to be finalized with completion of the SHINE trial. Including Ultra in the composite microbiological reference standard risks inclusion bias; however, we feel that this is justifiable, as Ultra is a highly specific test *in vitro* (98.7%) and is currently being used as a WHO-endorsed standard diagnostic test in this population. In Ugandan children with a high baseline disease prevalence, symptoms consistent with PTB, and anti-TB treatment given, a positive DNA-based test is less likely to represent a false positive than in other populations. Ideally, in our opinion, samples positive only by Ultra could be further tested for presence of mRNA or 16S rRNA to further investigate the presence of replicating or live bacteria. Furthermore, prolonged storage of specimen in a freezer may have affected the performance of Ultra, and this requires further investigation.

In conclusion, this study found Ultra to be more sensitive than Xpert for the detection of M. tuberculosis in Ugandan children, which represents an important advance for a condition which has posed a diagnostic challenge for decades. Ultra is likely to be the best initial test for suspected childhood PTB, but there is a still a role for culture in testing those for whom one or more sputum samples are Ultra negative. Culture-based rifampin susceptibility also remains important in low-bacillary-load samples (trace category positive) for which Ultra is unable to determine the status of rifampin drug susceptibility and reports “indeterminate rifampin resistance.” Further prospective diagnostic studies in which Ultra is performed in real time and in settings with different TB burdens would provide further information about its diagnostic performance.
